# Clinicoradiological Correlation of Infarct Patterns on Diffusion-weighted Magnetic Resonance Imaging in Stroke

**DOI:** 10.7759/cureus.2260

**Published:** 2018-03-02

**Authors:** Zainab Hussain, Kiran Hilal, Muhammad Ahmad, Zafar Sajjad, Raza Sayani

**Affiliations:** 1 Department of Radiology, The Aga Khan University Hospital, Karachi.; 2 Department of Internal Medicine, The Aga Khan University Hospital, Karachi.

**Keywords:** ischemic stroke, infarction, cortical infarction, barthel index, pattern, diffusion weighted imaging

## Abstract

Diffusion-weighted magnetic resonance imaging (DW-MRI) represents a major advance in the early diagnosis of acute ischemic stroke. It can detect edema due to ischemia in the brain tissue. It not only establishes the presence and location of ischemic brain injury but also a relatively new concept is the determination of infarct patterns seen on diffusion imaging and its clinical correlation.

Objective

To determine the frequency of various infarct patterns and their relationship with functional outcome of the patient.

Materials and methods

A total of 108 patients with acute stroke were enrolled by purposive sampling. Magnetic resonance imaging (MRI) was obtained with departmental protocol and diffusion-weighted sequences. The clinical data was collected from medical records and functional outcome was assessed at the time of admission using Barthel Index (BI) which was dichotomized into poor and favorable outcomes. The radiological data was collected and three infarct patterns (cortical, subcortical, and territorial infarcts) were recorded from diffusion-weighted images. Association of other risk factors such as age, gender, diabetes, hypertension (HTN), hyperlipidemia, and smoking were also evaluated.

Results

Amongst the three infarct patterns, subcortical infarcts were noted with the highest proportion of 62% (67/108). The highest proportion of territorial infarcts (78.6%) was significantly associated with a poor outcome in comparison to cortical and subcortical infarcts. Cortical infarcts (61.5%) were significantly associated with good outcomes followed by subcortical and then territorial infarcts (p-value < 0.002). Amongst the risk factors, HTN was found to be highly prevalent followed by diabetes mellitus (DM).

Conclusion

Subcortical infarct pattern was the most common, followed by territorial and cortical infarct. The highest proportion of infarct pattern with good outcomes was seen with cortical infarcts followed by subcortical and then territorial infarct pattern. HTN and coronary artery disease (CAD) were the effect modifiers showing significant association with poor outcomes.

## Introduction

Stroke is one of the major causes of disability and death worldwide. While the developed world has recognized the disease burden and it risk factors in countries such as India, Pakistan, Bangladesh, and Sri Lanka, there is a constant rise in mortality, with the increasing incidence of hypertension (HTN) being the primary cause [1]. Pakistan is predicted to be the fourth most populous country with diabetes mellitus (DM) by 2020 [2]. And every third urban Pakistani above 45 years is diagnosed with HTN [3]. Due to the lack of sufficient economic resources and health awareness, despite timely diagnosis of hypertension, uncontrolled disease is prevalent. The Pakistan Stroke Society gives an estimated incidence of 250 per 100,000 which means that there are 350,000 new stroke cases per year [2].

The mainstay for the diagnosis of stroke has been conventional computed tomography (CT) scan and magnetic resonance imaging (MRI) [4]. However, with advances in radiology, diffusion-weighted MRI has emerged as the "gold standard" for stroke diagnosis. Diffusion-weighted images are very sensitive and specific for the detection of hyperacute and acute infarctions, with a sensitivity of 88%-100% and a specificity of 86%-100% in detecting infarcted tissues as early as one to two hours after onset [5]. Another new concept is the identification of various infarct patterns which are based on the involvement of brain parenchyma and are broadly classified as cortical, subcortical, and territorial [6]. Multiple studies have depicted a relationship between the etiology and infarct pattern. Therefore its association with the clinical outcome is also of significant value in clinical studies [7].

Many scales have been devised for the evaluation of clinical outcome in stroke patients [8]. The National Institute Health Sciences Scale is a validated tool for evaluation; however, due to its technicality, it is difficult to implement and therefore scales assessing the functional outcome of patients such as the Modified Rankin score and Barthel index (BI) are used which are easier to implement and provide knowledge regarding the daily activity and functional ability of the patient [9].

The purpose of this study is to determine the frequency of various infarct patterns and their relationship with functional outcomes of the patient.

## Materials and methods

The cross-sectional study was conducted at the Department of Radiology for six months. Stroke patients were sampled by non-probability purposive sampling. The sample size was calculated on the World Health Organisation (WHO) software version of sample size. The independent variables consisted of age, gender, diabetes, HTN, hyperlipidemia, smoking, and the infarct pattern that was seen and included in the report on MRI images.

The dependent variables consisted of functional outcomes of stroke assessed by the BI at the time of admission [6-7]. The scale was categorized into two categories as good outcome (score of 60) and bad outcomes (score of < 60) on the BI. Informed consent was obtained from the patients on the consent form. The data was collected in a structured questionnaire. 

All images were interpreted on picture archiving computer systems (PACS) by the primary investigator and the supervisor. Imaging protocols were as follows: axial T1-weighted (W), axial T2-W, sagittal T2-W, Coronal Flair as well as diffusion-weighted imaging using b values of b0, b500, and b1000. Apparent diffusion coefficient (ADC) images were also evaluated for confirmation of acute stroke.

**Figure 1:**
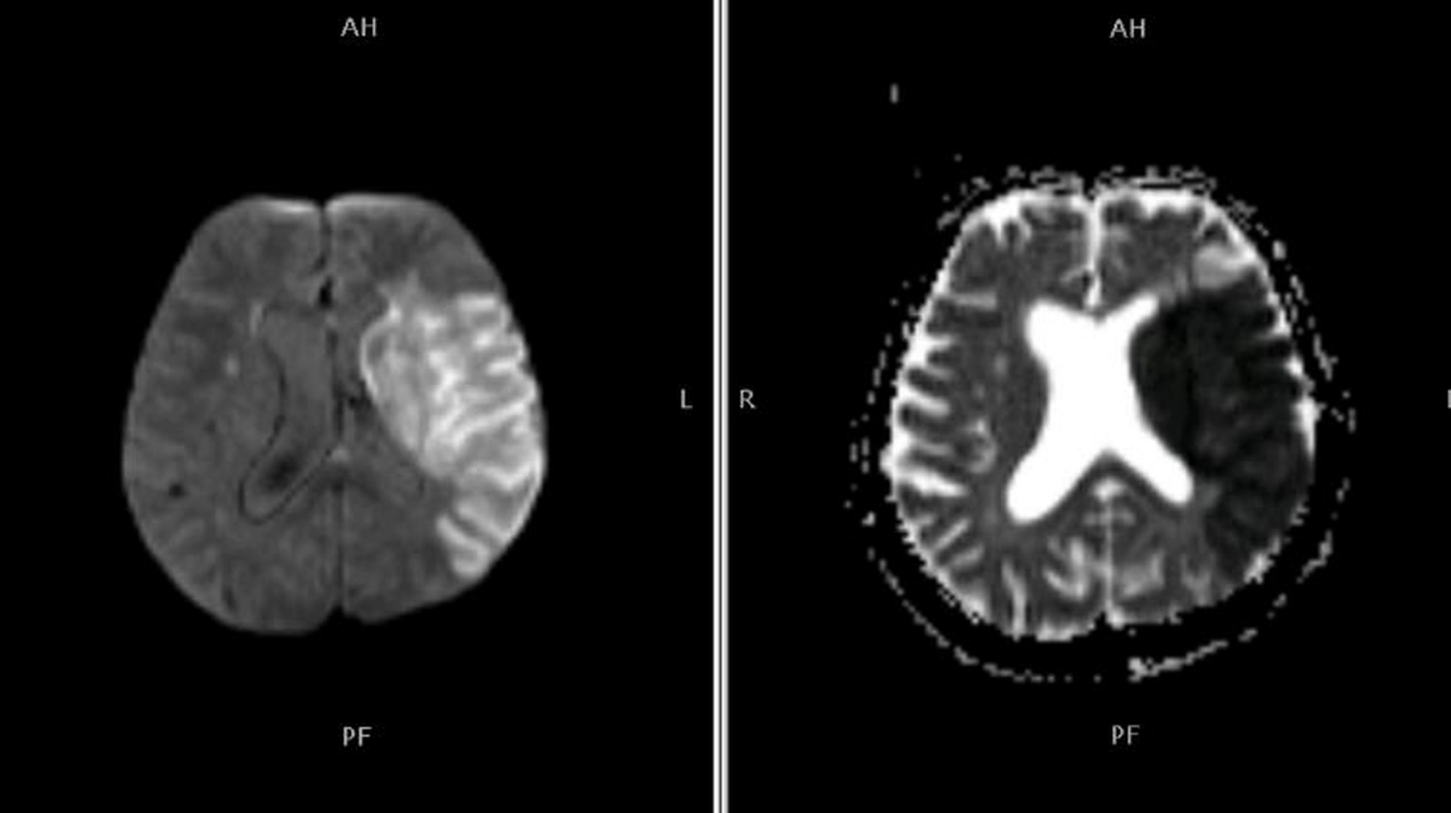
Territorial infarct pattern Diffusion-weighted imaging (DWI) shows bright DWI and low apparent diffusion coefficient (ADC) indicating acute territorial infarct. Patient had a significantly poor outcome and had diabetes as well as hypertension.

The infarct patterns were identified on the MRI. They were divided into three categories which are as follows:

Territorial: this indicates that the area affected was the portion of the brain that is supplied by that artery and includes various structures such as the cortex, deep white matter, and basal ganglia showing as a bright signal (Figure 1).

**Figure 2:**
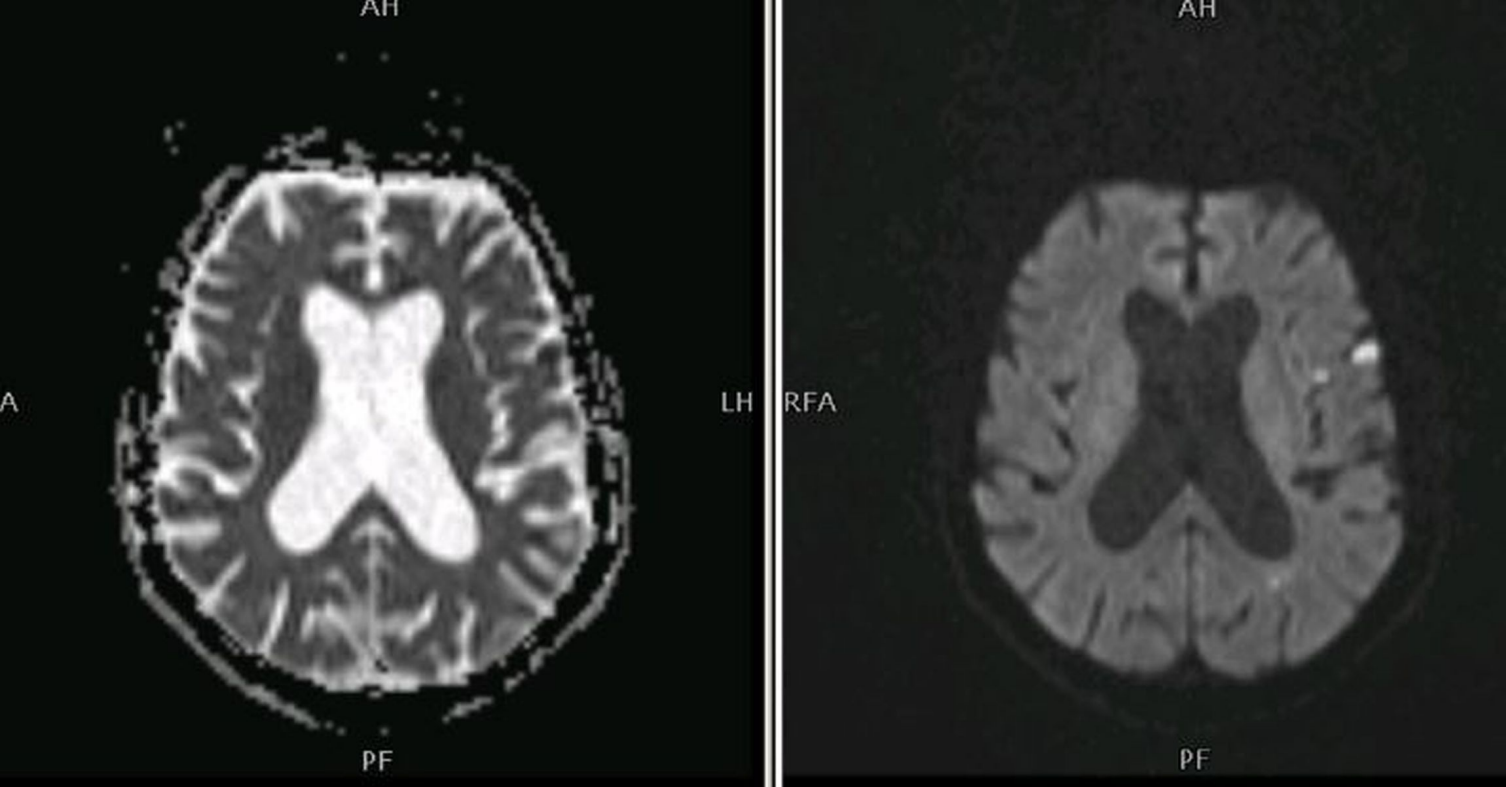
Cortical infarct pattern Bright diffusion-weighted imaging (DWI) and low apparent diffusion coefficient (ADC) images showing a small cortical stroke. The patient was hypertensive and had a score of more than 60 on the Barthel index.

Cortical: this indicates the involvement of only the peripheral portion of the brain which is known as the cortex again showing as a bright signal (Figure 2).

**Figure 3:**
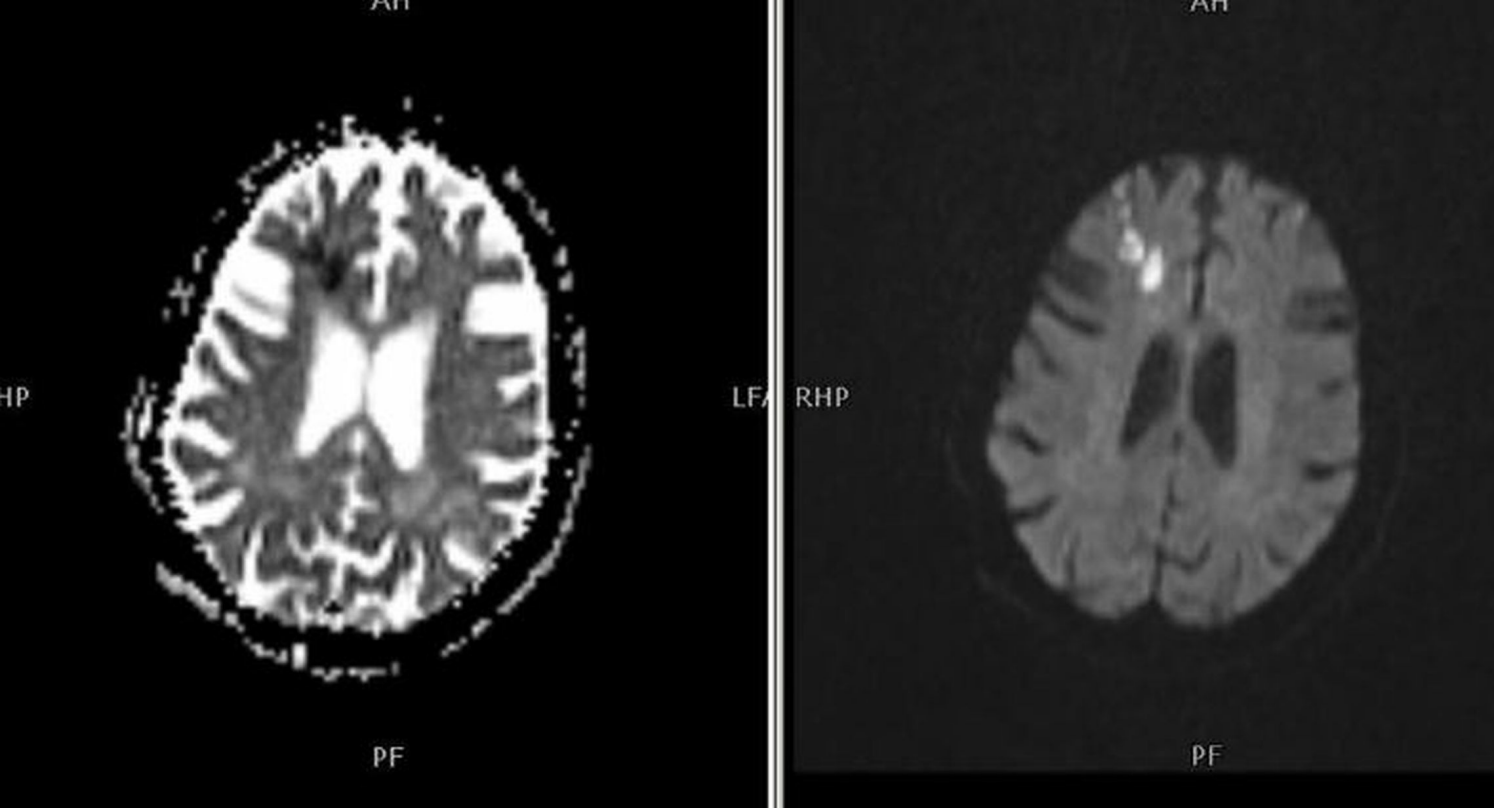
Subcortical infarct pattern Patient had poor outcome risk factors of hypertension, coronary artery disease, and dyslipidemia.

Sub-cortical: this is the area that lies beneath the cortex and includes deep white matter nuclei and basal ganglia representing disease; the cortex is spared (Figure 3).

All patients above 18 years, with the clinical diagnosis of stroke, with weakness or inability to move the limbs, loss of speech, loss of vision, or walking disturbances and confirmed by the MRI scan as ischemic stroke, were included. Strokes due to intracranial hemorrhage or patients whose imaging findings reveal no imaging diagnosis of stroke were excluded.

Data analysis was performed using SPSS 20 (IBM Corp., Armonk, NY, US). A p value of <0.005 and confidence interval of 95% was considered significant. The association of confounding effects of variables such as age, diabetes, HTN, hyperlipidemia, and smoking was also assessed with respect to infarct pattern and functional outcome.

## Results

A total of 108 patients were included in the study. The age ranged from 33 to 101 with the mean age of patients at 63.8 ± 13.9 years. There were 70 males and 38 females included in the study. The frequency of the infarct patterns calculated was 14 cortical infarcts, 67 subcortical, and 27 territorial infarcts confirmed on MRI. The outcome of patients was evaluated on the BI and categorized into favorable and poor outcome with a score of 0-59 as poor and 60-100 as favorable. Out of 108 patients, 56 had a poor outcome while 52 had favorable scores. 

The presence of risk factors was also accounted for to control the effect of confounding variables. HTN was the most prevalent risk factor with 74 patients as hypertensive. This was followed by DM which was present in 52 patients and hyperlipidemia which was seen in 51 patients. Coronary artery disease (CAD) was seen in 23 patients and 23 patients gave a history of smoking (Table 1).

**Table 1:** Distribution of variables HTN: hypertension; DM: diabetes mellitus; CAD: coronary artery disease; DLD: dyslipidemia; SM: smoking; BI: Barthel Index.

	Male (70)	Female (38)	Total (108)
Infarct pattern			
Cortical	9	5	14
Subcortical	41	26	67
Territorial	19	8	27
Total number of infarcts			108
HTN	40	34	74
DM	31	21	52
CAD	15	8	23
DLD	29	22	51
SM	22	1	23
Poor outcome (BI < 60)	34	22	56
Favorable outcome (BI ≥ 60)	35	17	52

Amongst the three infarct patterns, there was a significant difference in the outcome with a p-value of 0.004. Out of 28 territorial infarcts, 22 (78.6%) were associated with a poor outcome. This was followed by subcortical infarcts which constituted 43.3% (29/67) of poor outcome patients. Only 37.5% of patients with cortical infarcts (3/8) had a poor outcome (Table 2).

**Table 2:** Distribution of infarct patterns with outcome BI: Barthel Index

	Poor outcome (BI <60)	Favorable outcome (BI>60)	Total
Cortical	5 (38.5)	8 (61.5)	13 (100%)
Subcortical	29 (43.3)	38 (56.7)	67 (100%)
Territorial *	22 (78.6)	6 (21.4)	30 (100)

In the subset analysis, no significant difference was seen in the proportions of bad outcome in the comparison of cortical versus subcortical infarct pattern (43.3% vs. 37.5% (p-value 0.5)). However, a significant difference between the proportions of territorial versus cortical infarct pattern, and territorial versus subcortical infarct pattern was seen with adverse outcomes (p-values = 0.008 and 0.002) respectively. The highest number of patients with favorable outcomes was seen with cortical infarcts in which 61.5% of the infarcts were associated with favorable outcomes. This was followed by patients with subcortical infarcts; 56.7% of subcortical infarcts were associated with favorable outcomes. The least number of patients with good outcomes was seen with territorial infarcts; only 21.4% of them were associated with a good outcome. In the comparison of cortical versus subcortical infarct pattern, no significant difference was seen in patients with favorable outcomes (p-value <0.005). However, there was a significant difference between the outcomes in patients with cortical versus territorial and subcortical versus territorial infarct (p-values = 0.008 and 0.002) respectively.

The effect of HTN, DM, hyperlipidemia, and CAD was evaluated with functional outcome. Amongst all these variables, the presence of HTN was seen in 74 patients and was associated with poor outcomes in 54 patients which was found to be significant with a p-value of 0.02. Although only 23 patients had CAD, significant association with poor outcome was seen in 11 of these with p-value 0.02. However, in a large number of patients who did not have CAD, poor outcome was seen in 39 patients, which were also clinically significant (p-value of 0.019). The presence of DM was seen in 52 patients and did not show significant association with the outcome (p-value 0.106). However, the absence of DM was significantly associated with good outcome; p-value of 0.02. Smoking and hyperlipidemia were not found to have significant association with adverse outcomes. They were present in only 23 patients and 51 patients with p-values of 0.32 and 0.19 respectively.

## Discussion

Stroke has been reported to be the second largest cause of death worldwide and the third most common cause in first world countries. There is no cost-effective curative therapy for stroke and the bulk of public health initiatives are focused on prevention [8].

A new approach for evaluation of stroke is being used where the actual appearance of an infarct on radiological images (particularly diffusion-weighted images) is used. This is known as the evaluation of infarct patterns and is being used to recognize the etiology of stroke in different populations. Studies are also being conducted to evaluate the outcome. In this study, prospective evaluation has been performed to identify the proportions of topographical appearances of infarct in the brain parenchyma and their association with functional outcomes of the patients. We also tried to evaluate the association of known risk factors of stroke in these patients with different infarct patterns and functional outcomes.

It was found that the highest frequency of infarct patterns was the subcortical type occurring in 62.6% of the patients. This was followed by territorial infarct which was seen in 24.3% of the patients and then cortical infarcts seen in 13.1% of the patients. The proportion of infarcts that were seen in the subcortical group was significantly higher than that reported in another study conducted in South Korea where a total of 12.1% infarct were subcortical which were further divided into cortical border zone and subcortical border zone [7]. In another study, the proportion of cortical and subcortical infarcts was 83.7% while territorial infarcts accounted for 17.3%. In this study, another different classification was used for cortical and subcortical infarcts [9]. However, the proportion of subcortical infarcts is somewhat closer to what is seen in our sample. The classification that is used in the current study was also used in a study conducted in Korea in 2003 where 67.7% of the infarcts were subcortical and 12% were territorial [6]. This again shows a similarity to the prevalent proportions in our study suggesting that subcortical patterns are the most prevalent patterns seen. The proportion of cortical infarcts mentioned was 6.5%.

In all of the above-mentioned studies, infarct patterns and their associations with stroke subtypes and etiologies have been attempted. However, we did not carry out an evaluation of the magnetic resonance angiography (MRA) and other TOAST criteria (Trial of Org 10172 in Acute Stroke Treatment) for the diagnosis as we were only evaluating outcomes of the infarct patterns. Another aspect that was not evaluated in their studies was the gender difference between different infarct patterns. Overall, we had an abundance of male patients with stroke; 70 (64.5%) males and 38 females (35.5%). Despite this, no significant statistical difference was seen amongst the prevalence of any one infarct pattern over the other in either gender. This is indicative of the fact that stroke appears to be more of a disease afflicting the male gender irrespective of infarct pattern and etiology.

The outcome of this study has been evaluated with the BI. Various studies in the literature have used this index as well as many others for the evaluation of stroke outcome such as the National Institutes of Health Stroke Scale (NIHSS) and modified Rankin Scale [10-11]. The NIHSS has been used for evaluation of a neurological deficit and has been reported to be sensitive in assessing treatment efficacy. However, a ceiling effect of falsely poor outcomes is noted as many commands cannot be followed by stroke patients [12]. Implementation of this scale was not feasible as it required a full-time trained physician for conducting the exam. Various studies have now advocated the use of functional scales for assessing patient disability. This has been further assessed with a comparison of BI with MRS where very good internal consistency of the BI was noted [13].

In this study, a significant difference between the outcomes was evaluated between the cortical and territorial infarcts as well as the subcortical and territorial subgroup with a significant association of territorial infarcts with poor outcome. No statistically significant difference was seen in the cortical versus subcortical pattern for both favorable and poor outcomes. Multiple studies that have been conducted have primarily compared outcome scales with the etiological subtype of stroke. In the study by Lee et al., correlation of neurological outcome by NIHSS was seen with etiology of stroke and the outcome was seen to be worse in patients with internal carotid artery atherosclerosis as seen on MRA images. In the study conducted by Yong et al., a comparison was made between subtypes of subcortical infarct internal border zone and cortical border zone infarcts and worsening outcome was seen in internal border zone infarct [14]. In another study by Lee et al., comparison of infarct patterns was made in middle cerebral artery (MCA) disease which revealed that worse neurological outcome with NIHSS was seen in patients with cardiogenic source of embolism rather than atherosclerotic disease and the infarct pattern seen with the embolism group was of the cortical and territorial type [6]. These results are somewhat similar to ours in which 77.3% of territorial infarcts are associated with BI score < 59 indicating adverse outcome with the territorial pattern.

There are multiple risk factors that have been described with stroke in the literature [15]. In a local study conducted by Syed et al., the frequency of ischemic stroke subtypes are described. They found HTN as the most prevalent risk factor (66.2%) followed by DM in 41.5% patients [16]. This is similar to what we saw in our study where the highest proportion of HTN was 68.5%. In the study conducted by Lee in 2004 for determining etiology of MCA stroke, it was seen that significant association between DM and HTN was noted in the group with atherosclerotic MCA disease [5]. In our study, we could not establish a significant association of DM with outcomes. Subcortical infarct pattern had the highest proportion of all risk factors as seen in Table1. However, significant association with adverse outcome was only seen with HTN and CAD.

Multiple studies have shown that HTN is the single most important risk factor for stroke and trials have reported that significant reduction in stroke cases have occurred with effective antihypertensive treatment. DM is another important risk factor that has shown an independent risk of atherosclerosis with brain infarctions. Case-control studies of stroke patients and prospective epidemiological studies have confirmed an independent effect of DM with a relative risk of ischemic stroke in persons with DM from 1.8 to 3.0. In the Honolulu Heart Program, those with DM had twice the risk of thromboembolic stroke than persons without DM that was independent of other risk factors [17]. The duration of DM was not accounted which leads to the onset of complications.

In our study, CAD was found to be significantly associated with poor outcomes. Myocardial disease has long been recognized as a risk factor for stroke. In the Framingham Study, when multivariate analysis was used, the risk of stroke was increased twofold by coronary heart disease, threefold by electrocardiographic left ventricular hypertrophy, and threefold to fourfold by cardiac failure [18]. In a separate analysis at Framingham, left ventricular mass assessed by echocardiography was also predictive of stroke in follow-up. The other risk factors for hyperlipidemia and smoking were not found to be significantly associated with any particular infarct pattern or the adverse outcome. However, their potential as effect modifiers has been established in multiple trials. While hypercholesterolemia is an important modifiable risk factor for CAD, the link to ischemic stroke is still uncertain [19-20].

This study had a few limitations. Only one scale was implemented for the evaluation of outcomes and the neurological deficit was not accounted for. Only one racial group was included in the study. The distribution of infarct patterns was not uniform with an excess of the subcortical types. Other risk factors such as physical activity and obesity were not accounted for as measuring body mass index (BMI) was not possible for all patients.

## Conclusions

Diffusion-weighted MRI is a noninvasive modality that is used for the diagnosis of acute stroke. Infarct patterns can be reliably used to predict functional outcomes in patients. In this study, we found the highest frequency of subcortical infarcts followed by territorial and cortical infarct patterns. The highest proportion of infarct pattern with good outcomes was seen with cortical infarcts followed by subcortical and then territorial infarct pattern. HTN and CAD were the effect modifiers showing significant association with poor outcomes.
